# Supplementary material for: Complexome profiling of the Chlamydomonas psb28 mutant reveals TEF5 as an early PSII assembly factor
Source: Plant Cell. 2025 Mar 17;37(6):koaf055. doi: 10.1093/plcell/koaf055 (PMC12164590; doi:10.1093/plcell/koaf055)
Supplement: koaf055_Supplementary_Data [file koaf055_supplementary_data.zip › psb28_interactive.zip!/plots/intensity/qualityPlots/peptides/_Cre08.g374950.t1.1.html]

### Cre08.g374950.t1.1

Trivial name:   
SeqCoverage [median]: 4.90  
Mass: 76.36 kDa  
  
 

| Sequence | MissedCleavage | Mass [Da] | wtIntensity | muIntensity | Score |
| --- | --- | --- | --- | --- | --- |
| AVGALVEETR | 0 | 1043.56 | 0.00 | 1109.21 | 92.44 |
| QLAASIPTTTIR | 0 | 1270.72 | 0.00 | 342.87 | 52.79 |
| TATGGAGASAPASSR | 0 | 1260.61 | 0.00 | 399.84 | 54.70 |
|  |  |  | 0.0 | 1851.9 |  |
